# How much time do nurses have for patients? a longitudinal study quantifying hospital nurses' patterns of task time distribution and interactions with health professionals

**DOI:** 10.1186/1472-6963-11-319

**Published:** 2011-11-24

**Authors:** Johanna I Westbrook, Christine Duffield, Ling Li, Nerida J Creswick

**Affiliations:** 1 Centre for Health Systems and Safety Research, Australian Institute of Health Innovation, Faculty of Medicine, University of New South Wales, Sydney, 2052, Australia; 2 Centre for Health Services Management; and WHO Collaborating Centre for Nursing, Midwifery and Health Development, Faculty of Nursing, Midwifery and Health, University of Technology, Sydney, 2007, Australia

## Abstract

**Background:**

Time nurses spend with patients is associated with improved patient outcomes, reduced errors, and patient and nurse satisfaction. Few studies have measured how nurses distribute their time across tasks. We aimed to quantify how nurses distribute their time across tasks, with patients, in individual tasks, and engagement with other health care providers; and how work patterns changed over a two year period.

**Methods:**

Prospective observational study of 57 nurses for 191.3 hours (109.8 hours in 2005/2006 and 81.5 in 2008), on two wards in a teaching hospital in Australia. The validated Work Observation Method by Activity Timing (WOMBAT) method was applied. Proportions of time in 10 categories of work, average time per task, time with patients and others, information tools used, and rates of interruptions and multi-tasking were calculated.

**Results:**

Nurses spent 37.0%[95%CI: 34.5, 39.3] of their time with patients, which did not change in year 3 [35.7%; 95%CI: 33.3, 38.0]. Direct care, indirect care, medication tasks and professional communication together consumed 76.4% of nurses' time in year 1 and 81.0% in year 3. Time on direct and indirect care increased significantly (respectively 20.4% to 24.8%, P < 0.01;13.0% to 16.1%, P < 0.01). Proportion of time on medication tasks (19.0%) did not change. Time in professional communication declined (24.0% to 19.2%, P < 0.05). Nurses completed an average of 72.3 tasks per hour, with a mean task length of 55 seconds. Interruptions arose at an average rate of two per hour, but medication tasks incurred 27% of all interruptions. In 25% of medication tasks nurses multi-tasked. Between years 1 and 3 nurses spent more time alone, from 27.5%[95%CI 24.5, 30.6] to 39.4%[34.9, 43.9]. Time with health professionals other than nurses was low and did not change.

**Conclusions:**

Nurses spent around 37% of their time with patients which did not change. Work patterns were increasingly fragmented with rapid changes between tasks of short length. Interruptions were modest but their substantial over-representation among medication tasks raises potential safety concerns. There was no evidence of an increase in team-based, multi-disciplinary care. Over time nurses spent significantly less time talking with colleagues and more time alone.

## Background

Central to the care of patients and the satisfaction of nurses is the amount of time they are able to spend with patients. Time nurses spend in direct care activities has been identified as a determinant of better patient outcomes and fewer errors [1-3]. Patient satisfaction is also related to the amount of direct care received [4]. Qualitative studies reveal clinicians' satisfaction is associated with time spent in clinical work [5] and that clinicians are dissatisfied with the amounts of 'excessive paperwork' and 'wasted time' spent locating other professionals [6], documents or equipment [7]. Thus initiatives which are effective in allowing clinicians to shift their time to direct care are likely to produce improvements in health outcomes, and patient and health professionals' satisfaction, which may also impact upon improved staff retention [3,8].

Two priority areas of health reform internationally are to improve the productivity of the workforce to address growing service demands[9-11]; and increase the level of inter-disciplinary care and communication to enhance the quality and safety of services[12,13]. Ratios of nurses to patients on general wards is a frequently applied metric, yet reveals little about the ways in which this nurse time resource is deployed to support patient care. If a primary objective is to ensure nurses spend sufficient time with patients in direct care and are engaged in inter-disciplinary care provision, then direct measures of these are required. There are surprisingly few baseline data about how nurses distribute their time, or the extent to which nurses engage with other health professionals in care provision against which the effectiveness of strategies can be tested. This absence of evidence also hinders debate about what are the most appropriate and effective levels of direct care provision.

We aimed to quantify how different classifications of nurses on hospital wards distribute their time across tasks, their time in individual tasks, and the extent to which they engage with other health care providers. We then assessed how these patterns of work changed over a two year period.

## Methods

### Setting

The study was conducted in two wards in a 400-bed major public hospital in Sydney in 2005/2006 (year 1) and then repeated in 2008 (year 3). Both wards used paper medical records and medication charts, but the hospital had a computerised order entry system for ordering of diagnostic tests and viewing of results as well as ordering of diets, transport, porters and allied health consultations. Ward nurses included in the study worked shifts of 8.5 hours in length. In year 1 both wards used a patient allocation model where each nurse was assigned 3-4 patients. In year 3 both wards used a team allocation model where a team of three nurses were assigned 10-12 patients.

### Study design and procedures

We used a prospective observational study design to identify changes in patterns of nurses' work on two general medical and surgical wards. The study wards had an average of 28 beds and included the specialty areas of respiratory and renal/vascular medicine.

The study was conducted over 41 months with data collected between July 2005 and March 2006 and between August 2008 and December 2008. All nurses on the two wards were invited to participate in the study via information sessions followed by a direct approach. In total 57 nurses (approximately 80% participation) were observed for a total of 191.3 hours between the hours of 7:00 and 19:00 on weekdays. Twenty-seven nurses were observed for 109.8 hours in year 1, and 30 were observed for 81.5 hours in year 3.

Rosters (schedules) from each ward were used to calculate the full time equivalents (FTEs) for each nurse classification (enrolled nurse [one year vocational preparation], registered nurse-new graduate, registered nurse 2 to 4 years, registered nurse 5+ years, clinical nurse specialist). Representative sampling was used to determine the number of minutes that participants needed to be observed for each hour of the day for each classification of nurse. Following signed consent, nurses were assigned a study identification number, and demographic information regarding their age, nurse classification, and length of experience was collected. Nurses were given no prior warning of observation periods. Observers randomly allocated participating nurses to a list for each observation session according to the sampling strategy. If a nurse at the top of the list was not working that day, observers selected the next one on the list.

The Work Observation Method by Activity Timing (WOMBAT) method was applied [14-16]. This comprises a modified time and motion approach which includes a multi-dimensional work task classification system incorporated into a handheld computer (personal digital assistant-PDA). The method collects information about 10 broad, mutually exclusive work categories. Table 1 describes each of these ten categories and sub tasks. This classification was developed following extensive observations and pilot testing described previously [17,18]. The method has been applied in Australian studies of doctors on hospital wards [15], in an emergency department [19], and hospital pharmacists [20]. Most recently the technique was validated in Canadian studies of intensive care clinicians [16,21]. The observers shadowed nurse participants for an average of one hour blocks, recording data on all work tasks performed using the PDA. For each task the data collector recorded with whom the nurse completed the task, the information tools used and any interruptions to work (defined as ceasing a task in order to respond to an external stimuli) or tasks completed in parallel (multi-tasking). Each task is automatically time and date stamped when entered into the PDA.

**Table 1:** Task and information tool definitions

Work Task	Definition
Direct Care	Tasks directly involved with patient care, eg direct communication with patient &/or family, bathing, applying dressings, nursing procedures etc.
Indirect Care	All tasks indirectly related to patient care, eg reviewing results, planning care, washing hands, reviewing documentation, returning equipment
Medication Tasks	All tasks associated with medication, includes preparation, administration, documentation, discussion & clarification
Documentation	Documentation (paper and electronic), excludes medication documentation
Professional Communication	All non-medication related communication with another health professional includes ward & patient handover. Excludes medication related discussion communication
Ward Related Activities	Ward activities, includes coordinating beds & staffing
In Transit	Time between tasks and between patients. Excludes movement between patients in a shared room and movement within a single room
Supervision	Supervising others, including students
Social	All non work communication, eg meal/tea breaks, personal calls
Other	Any other task not included above
Information Tools Used	
Permanent Record	Tasks involving the writing/reading of information in a patient's permanent paper medical record, including: progress notes, request forms, medication chart, observation chart, nursing care plans
Paper	Tasks involving writing on temporary paper notes, eg bedlist, handover sheet, little notebook
Desktop Personal Computer (PC)	Tasks involving the use of a computer for information searching, retrieval and documentation
Phone	Tasks involving using the telephone

When the participant nurse engaged with patients, visitors, or other health professionals, the nurse was asked to introduce the observer and seek permission to continue. Alternatively, the observer would identify themselves. Several dummy observation sessions were undertaken as part of the observer training process. This also allowed nurses to become accustomed to being observed. The study was approved by the human research ethics committees of the University of New South Wales and the study hospital.

### Observer training

All observers were clinically experienced registered nurses or medical doctors. Inter-rater reliability tests were performed with two data collectors simultaneously, but independently, observing a nurse and comparing data. Kappa scores[22] for task classification was > 0.89 throughout data collection indicating high levels of agreement between observers.

### Statistical Analysis

Descriptive statistics were calculated for average task length, number of tasks per hour and proportion of nurses' time the task consumed. This analysis is presented by task category, whether completed with other health professionals and/or patients, the information tool used, day of the week and nurse classification. Rates of interruptions and proportion of time multi-tasking were calculated. The 95% confidence intervals of the proportions of time spent on the different tasks were obtained using the large sample normal approximation. Comparisons between study periods, clinical roles and day of the week are presented and relevant comparisons were made using the t-test, with the level of significance set at P < 0.05. Data were analysed using SAS version 9.2 [23].

## Results

### Time spent with patients

In year 1 nurses spent 37.0% (95%CI 34.5, 39.3) of their time with patients and this did not change significantly in year 3 (35.7%; 95%CI 33.3, 38.0) (Table 2). During an average 8.5 hour shift this equates to approximately 3.1 hours per shift spent with patients.

**Table 2:** Time spent by nurses with other health professionals and patients

With whom	Period	Number of tasks	Mean task length (seconds)	Percentage* (%)	95% Confidence intervals	
**Alone**	Year 1	2630	41.37^#^	27.52	24.45	30.59
	Year 3	2288	50.48^#^	39.38	34.90	43.87
**Patient**	Year1	2394	61.01	36.95	34.55	39.34
	Year 3	1666	62.77	35.66	33.29	38.03
**Relative**	Year 1	401	59.69	6.05	5.27	6.84
	Year 3	282	57.45	5.52	4.71	6.34
**Nurse**	Year 1	2665	80.64^#^	54.36	49.05	59.67
	Year 3	2229	53.87^#^	40.95	36.93	44.97
**Doctor**	Year 1	327	44.19	3.66	3.26	4.05
	Year 3	240	43.37	3.55	2.96	4.14
**Allied Health**	Year1	83	55.67	1.17	0.88	1.46
	Year 3	67	34.48	0.79	0.60	0.98
**Pharmacy**	Year 1	31	46.23	0.36	0.20	0.52
	Year 3	35	40.49	0.48	0.37	0.60
**Health professional^**	Year 1	2976	76.57^#^	57.64	52.30	62.98
	Year 3	2518	52.34^#^	44.94	40.88	49.00
**Other**	Year1	416	51.87	5.46	4.82	6.10
	Year 3	290	38.52	3.81	3.18	4.44

*Percentage of observed task time. The categories of with who are not mutually exclusive (with the exception of 'alone'). ^Health professional includes nurses, doctors, allied health professional including pharmacists. # indicates a statistically significant change in year 1 and year 3 average task lengths(P < 0.05).

**Figure 1:**
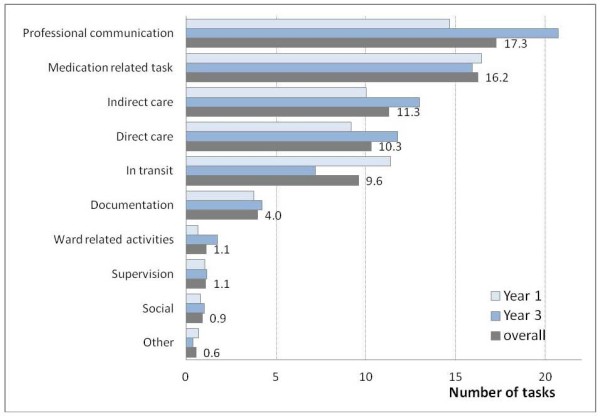
**Number and type of tasks completed by a nurse in an average hour by study year (Mean = 68.7 tasks/hour)**.

Overall, nurses completed 72.3 tasks per hour. Figure 1 shows the number and type of different tasks undertaken in an average hour. Professional communication and medication tasks were the most frequent.

### Proportion of time nurses spent in tasks, by nurse classification and changes over time

**Figure 2:**
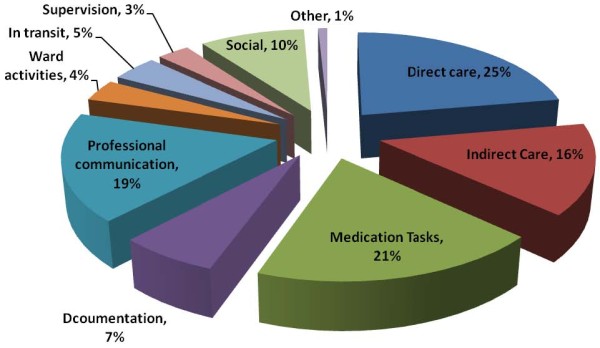
**Proportion of time spent on different tasks in year 3**.

Direct care, indirect care, medication tasks and professional communication together consumed approximately 76% of nurses' time in year 1 and 81.0% in year 3 (Table 3 and Figure 2). In the two year period the proportion of time spent on direct (20.4% to 24.8%, P < 0.01) and indirect care (13.0% to 16.1%, P < 0.01) increased significantly (Table 3). The proportion of time spent on medication tasks did not change, and that spent in professional communication (24.0% to 19.2%, P < 0.05) and documentation (9.7% to 7.3%, P < 0.05) decreased. Time spent in transit was the only other task type which significantly changed, falling from 7.4% to 4.6% of nurses' time (P < 0.01) (Table 3).

**Table 3:** Number, proportion and average task times in year 1 and year 3 by task type

Task	Period	Number of tasks	Total time spent on task (hrs)	Mean task length (seconds)	Number of tasks per hour	Percentage of total time	95% Confidence intervals	
Direct care	Year 1	1009	22.4	80	9.2	20.4	18.3%	22.4%
	Year 3	958	20.2	76	11.8	24.8	22.7%	26.9%
Indirect care	Year 1	1098	14.2	47	10.0	13.0	11.6%	14.3%
	Year 3	1059	13.1	45	13.0	16.1	14.3%	18.0%
Medication tasks	Year 1	1808	20.9	42*	16.5	19.0	18.1%	19.9%
	Year 3	1296	17.0	47*	15.9	20.9	18.7%	23.0%
Documentation	Year 1	416	10.7	92*	3.8	9.7	7.7%	11.7%
	Year 3	342	6.0	63*	4.2	7.3	6.4%	8.2%
Professional	Year 1	1612	26.3	59*	14.7	24.0	20.7%	27.2%
communication	Year 3	1688	15.6	33*	20.7	19.2	17.1%	21.3%
Ward activities	Year 1	72	2.9	144*	0.7	2.6	1.7%	3.6%
	Year 3	143	3.2	80*	1.8	3.9	2.6%	5.2%
In transit	Year 1	1251	8.1	23	11.4	7.4	6.7%	8.0%
	Year 3	584	3.8	23	7.2	4.6	2.9%	6.4%
Supervision	Year 1	116	2.6	80	1.1	2.3	1.7%	3.0%
	Year 3	94	2.6	101	1.2	3.2	2.5%	4.0%
Social	Year 1	90	13.3	531	0.8	12.1	8.5%	15.7%
	Year 3	85	8.5	360	1.0	10.4	7.0%	13.9%
Other	Year 1	78	0.7	30	0.7	0.6	0.5%	0.8%
	Year 3	31	0.7	84	0.4	0.9	0%	2.0%

*Indicates significant change in year 1 and year 3 average task length times (P < 0.05)

**Figure 3:**
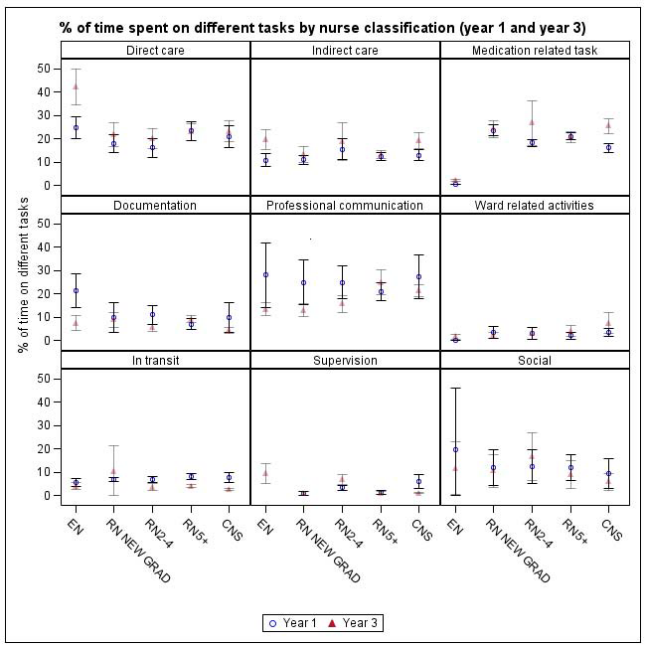
**Proportions of time spent in different tasks by nurse classification in Year 1 and Year 3**.

Task time distribution was similar for different nurse classifications with the exception of enrolled nurses who spent more time in direct care and less time in medication tasks and ward-related activities and no time supervising staff (Figure 3). This is consistent with their reduced clinical role compared to registered nurses.

### Average time spent on each task and changes over time

The average length of individual tasks ranged from 23 seconds (in transit) to 8.9 minutes (social) in year 1 (Table 3). In total, nurses changed tasks on average every 55 seconds. There were significant declines in average task lengths for three categories of tasks (documentation P < 0.005; professional communication P < 0.0001; ward related activities P < 0.05) and a significant increase in the average length of individual medication tasks (P < 0.05) (Table 3).

**Figure 4:**
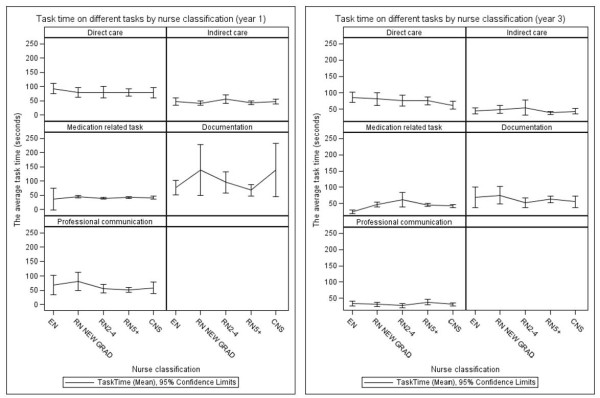
**Average length of tasks by nurse classification in Year 1 and Year 3**.

The mean length of tasks did not significantly differ by nurse classification for different tasks, and there was no evidence that less experienced nurses spent longer on tasks than experienced nurses (Figure 4). The proportion of time spent on professional communication significantly declined over time, from 24% to 19%, and the average time on each communication task almost halved (from an average of 59 seconds in year 1 to 33 in year 3) (Table 3). This change was also associated with a significant decrease in collaborative task completion by nurses (Table 2).

### Use of information tools to complete tasks and changes over time

Nurses spent 29% of their time completing tasks using a permanent paper record (eg a patient's medical record) and in year 3 this significantly increased by 6% to make up 35% of nurses' time (P = 0.0001, Table 4). Use of computers to complete tasks significantly increased over time from 1.1% to 1.9% (P < .005). This equated to around 1 task in every 100 completed involved the use of a computer (Table 4). Tasks completed with informal pieces of paper (eg post-it notes) and a phone did not significantly change over time.

**Table 4:** Number, average task length and overall percentage of tasks completed using specific information tools

Information tools used to complete task	Period	Number of tasks	Mean task length (seconds)	Percentage of observation time	95% Confidence intervals		Rate per 100 tasks
Permanent Record	Year 1	2292	50.05	29.02	26.86	31.17	30.4
Permanent Record	Year 3	2027	50.80	35.11	32.82	37.40	32.3
Paper	Year 1	302	125.36*	9.58	7.02	12.14	4.0
Paper	Year 3	282	79.23*	7.62	5.75	9.49	4.5
Desk PC	Year 1	43	98.47	1.07	0.74	1.40	0.6
Desk PC	Year 3	77	72.05	1.89	1.45	2.34	1.2
Phone	Year 1	135	62.72	2.14	1.79	2.50	1.8
Phone	Year 3	152	51.63	2.68	2.22	3.13	2.4
Nothing	Year 1	4869	58.41*	71.94	66.34	77.54	64.5
Nothing	Year 3	3912	51.76*	69.04	63.32	74.77	62.3

*Indicates significant change in year 1 and year 3 average task times (P < 0.01)

### Tasks by day of the week

**Figure 5:**
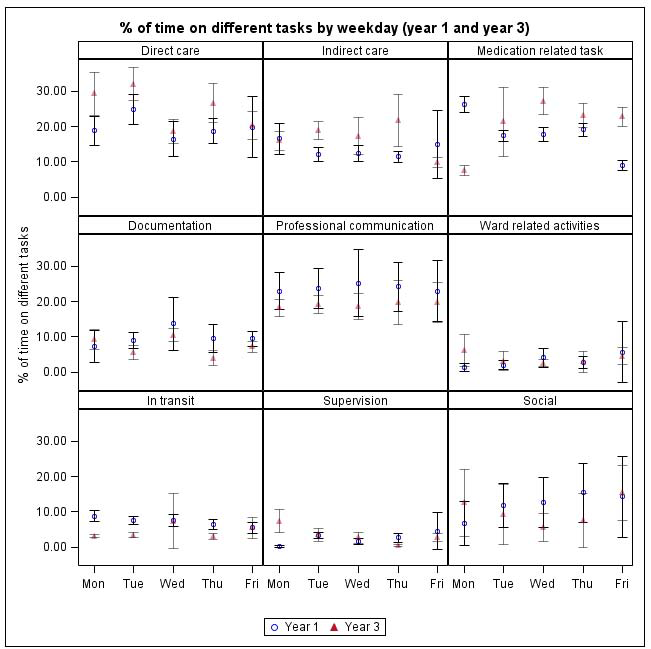
**Proportions of time spent on tasks by day and over time**.

There was limited variation in the proportions of time spent on different tasks by day of the week (Figure 5). The increases in time spent in direct and indirect care found in year 3 were distributed across the days of the week. One exception was medication tasks, which in year 1 consumed significantly more time on Mondays and less on Fridays. This position was reversed in year 3 (Figure 5).

### Multi-tasking and interruptions

In total the 57 nurses were observed for 191.3 hours and completed 13,830 tasks. For 5.8% of their time they were multi-tasking (ie completing two or more tasks in parallel). There were 374 interruptions recorded, a rate of one every 32 minutes. The highest proportion of interruptions occurred when nurses were undertaking medication tasks (27.3%, n = 102), followed by documentation (23%, n = 86). Multitasking was highly prevalent in medication tasks and occurred in 25% of all medication tasks performed. In 10.7% (n = 333) of medication tasks, nurses were concurrently conducting professional communication with a colleague.

### Collaborative work and time nurses spent alone

In year 1, nurses spent 57.6% of their time completing tasks with at least one other health professional (Table 2). Two years later nurses spent 44.9% of their time with other health professionals. Thus, as Table 2 shows, the amount of time spent completing tasks alone increased overtime from 27.5% to 39.4% in year 3. This was predominantly due to a decrease in time nurses spent undertaking tasks with other nurses (from 54.4% to 41.0%).

In year 3, when nurses completed tasks with others, they did so more quickly than in year 1. For example, the average time per task completed with another nurse in year 1 was 80.6 seconds and this fell to 53.9 in year 3 (P < .0001) (Table 3). There was no significant change in the percentage of tasks completed with others.

As Table 2 shows, the proportion of time that nurses spent with colleagues other than nurses was modest. For example, nurses spent 3.7% of their time with doctors in year 3. During an average nurse's shift of 8.5 hours this would equate to approximately 19 minutes per shift.

## Discussion

Nurses spent approximately one third of their time with patients and this did not change over time. A subset of this is the provision of direct care which significantly increased from 20% in year 1 to 25% in year 3. There are surprisingly few studies which have sought to quantify the amount of time nurses spend in direct care activities with patients and we have identified no study which has examined changes over time. Hendrich et al [24] in a study of multiple units at Kaiser Permanente in the United States reported an average of 19.3% of nurses' time (approximately 81 minutes per shift) was spent with patients. Using a diary method among 30 nurses in a Swedish hospital, Furaker [25] found around 38% of nurses' time was spent with patients. In year 3 the nurses in our study had moved to allocation of patients to nursing teams, but this appears to have had no effect on proportion of time spent with patients.

A central question is the extent to which this amount of time ensures safe care. Surveillance of patients by nurses has been identified as important to detect patients who are deteriorating. Research by Aiken and colleagues [1] has highlighted the relationship between nurse surveillance and patient safety. Surveillance relies on frequent interactions to be able to constantly monitor patients' conditions and provide opportunities to respond. On average we found each direct care task consumed approximately 80 seconds, and in an average hour nurses performed approximately 10 direct care tasks. However we were unable to assess how these tasks were distributed and this is likely to make a substantial difference to patient care. For example, 10 direct care tasks completed in quick succession leaves patients with no nurse contact for the remainder of the hour. However 10 tasks distributed evenly across the hour would provide much greater opportunity for surveillance. Further work is underway to develop methods to assess the sequencing of task distribution.

Few researchers have reported the amount of time which nurses spend on *individual *tasks. Nurses' work was characterised by a pattern of rapidly changing short tasks. Our findings are consistent with available evidence and suggest a general trend in the nature of nursing work on hospital wards. On average nurses in our study changed tasks every 55 seconds. Cornell et al[26] examined time spent in 29 task categories in a direct observational study on two wards and reported a similar high rate of task-switching with an average of 88 tasks per hour. We grouped work into 10 broad tasks and found a rate of 72 tasks per hour. Cornell et al[26] also found task length was short with only 5% of tasks lasting longer than two minutes. The implications of this rapid task changing activity in real-world settings have been underexplored. Experimental studies demonstrate that task-switching leads to increased errors and slower task performance [19,27-29]. One of the posited reasons for the slower performance when task-switching occurs is the cognitive effort required in reconfiguring the taskset which can involve both shifting attention to the new task while also inhibiting attention to a previous task [30]. Importantly these 'switch costs' have been shown to occur regardless of the participant's familiarity or training in the tasks performed [28]. The availability of preparation time prior to a task-switch has been shown in some cases to reduce switch costs [28,31]. The rapidity of task-switching found in the present study suggests nurses receive limited time to prepare for new tasks.

Our results demonstrate the reliance that nurses have on formal information sources to complete their work. Around 30% of their time involved tasks where formal paper records were used and this proportion increased over time. This was not due to greater demands on nurses to document information, as time spent in documentation did not increase over the two year period. The increased reliance on formal information sources may be a response to a decrease in access to information from other sources given that there was a significant fall in face to face professional communication and an increase in time nurses spent completing tasks alone. Use of computers constituted a very small amount of nurses' overall work, but increased over time. It is likely that with the introduction of greater computerisation, for example with computerised medication management and clinical documentation systems, time spent completing tasks with a computer will increase substantially.

A significant decline in time spent in transit was found. This may be related to greater access to computerised information sources and a decrease in seeking information face to face (as evidenced by the reduction in professional communication) both of which reduce the need to travel to obtain information. However without a focused study it is not possible to confirm the role of these factors in this result.

We found that nurses spent approximately 6% of their time multi-tasking and experienced approximately two interruptions per hour. Multi-tasking is an important component of health professionals' work and Australian doctors have been found to spend 20% of their time multi-tasking [15]. The majority of multi-tasking involved communication with patients or other health professionals and is a required feature of health care work which has rarely been quantified. Along with the results about interruptions it adds further evidence of the non-linear nature of clinical work.

Most concerning was that the highest proportion (27%) of all interruptions occurred during medication tasks. Further, 25% of medication tasks were undertaken in parallel with another task, most frequently professional communication. Kosit et al [32], in a study of interruptions in an emergency department, reported nurses were interrupted on average of 3.3 times per hour and that the highest proportion of interruptions (27%) occurred during medication tasks, as we found. Interruptions during medication tasks have been shown to be directly associated with the rate and severity of medication administration errors by nurses [33]. While nurses experienced rates of interruptions lower than their medical colleagues[15,19], their concentration during medication tasks suggests this task is at specific risk and interventions to reduce interruptions during this process are required [34,35].

No previous studies have followed nurses' work patterns over time. We found four broad categories of tasks consumed close to 80% of nurses' time (direct care, indirect care, professional communication, and medication tasks) in both periods. While the proportion of time in direct and indirect care increased, the amount in professional communication decreased significantly from 24% to 19% and the average time per communication task almost halved from 59 seconds to 33. While time spent in documentation decreased, but not significantly, (from 10% to 7% in year 3), the average time spent in each documentation task became significantly shorter (an average of 92 seconds to 33). Both these changes in the shortening of communication activities may be related to an increased reliance upon electronic communication and the greater use of clinical information systems reducing both the level of verbal communication required and the amount of documentation. While medication task time overall did not change, and continued to consume around 19% of nurses' time, the average medication task increased a small, though significant amount from an average of 42 seconds to 47 seconds per task. This may reflect the increased complexity of medication management among hospitalised patients requiring additional time, particularly in administration of medications. However whether this is an adequate amount of time is unclear. Research conducted by our team at this site on several wards, including the study wards, demonstrated high medication administration error rates and poor compliance with some medication administration procedures[33,36]. For example, direct observation revealed that in less than 50% of administrations did nurses correctly check patients' identification prior to drug administration[33]. The extent to which this reflects intentional deviation of practice or a response to time pressures is unknown. However there is good evidence that current practice is resulting in a high rate of medication administration errors [33,36].

The results provide little support for an increase in the amount of inter-disciplinary care or communication over time. Nurses experienced a dramatic increase in time spent completing tasks alone, from 28% (average 2.5 hours per shift) to 39% (average 3.5 hours per shift). This was largely due to a significant decline in the time spent with other nurses which fell from 54% to 41% of nurses' time. Interestingly, the results suggest that the requirement that certain tasks be completed with a colleague (the number of tasks completed with others did not change) may have led to nurses in year 3 completing joint tasks in significantly shorter times than in year 1. The average time for collaborative tasks with another nurse fell from 80.6 seconds to 53.9 seconds. This may reflect changes in nursing practice and/or compensation for a decrease in the availability of other nurses. For example, the move to team based allocation of patients may have led to nurses having a smaller pool of colleagues (ie those in their team) from whom to seek assistance in year 3. The amount of time nurses spent in professional communication significantly declined. There was little change in collaboration or communication with other health professionals which remained at very low levels. Cornell [26] also reported low levels of interactions between nurses and non-nursing colleagues making up around 2.8% of their time. On average nurses in our study spent approximately 3.6% of their time (18.4 minutes per shift) completing tasks with a doctor, while Cornell reported nurses in her US hospital spent only 0.5% (approximately 2.6 minutes per shift) of their time with doctors. Thus while the literature on the value of improved inter-disciplinary communication expands[37], our results suggest no evidence of increased interaction. Nurses on our study wards did not increase their level of engagement with other professionals. Further, the amount of time they worked collaboratively with other nurses substantially declined. This occurred in the context of both wards moving to a team-based nursing model. The impact of decreased collaborative task completion on care provision in terms of quality or efficiency is unknown and is worthy of consideration in future studies. While our study did not measure the content or quality of communication, the finding that the average length of a professional communication task almost halved between years 1 and 3 (from 59 seconds to 33) suggests little time is available for detailed information exchange about patient care.

### Limitations

The results reflect work patterns on two wards at one hospital and thus may not generalise to other hospitals with very different nursing practices. Our study examined weekday work. The results may not be representative of evenings or weekends. We used a direct observational approach, and while nurses may have changed their behaviours because they were being observed, the likelihood of dramatic change is low due to the extended length of the study, reducing the chance of sustained behavioural change on busy hospital wards. Observational studies of clinicians in-situ have suggested that the extent of behaviour change is minimal [17,38,39]. Strengths of our study include the longitudinal study design, consistency of methods and the data collection technique which accounted for multi-tasking, all of which have extended previous work in this area.

## Conclusions

The results present a picture of a fragmented pattern of work with increasingly rapid changes between tasks. Over time nurses experienced a shortening of the average length of key tasks such as professional communication and documentation. Nurses spent a significantly greater proportion of time alone and had significantly reduced contact with other nurses, while interactions with other health professionals did not change and remained low. Within this context nurses continued to spend around 37% of their time with patients. While the interruption rate was modest, at an average of two per hour, their distribution across tasks was not even. Medication tasks attracted 27% of all interruptions and 25% of these tasks were performed while nurses multi-tasked. Both these contextual factors are associated with increased risk of error. Little is known regarding the relationship between nurses' patterns of work and the quality of patient care. These results provide an indication of the ways in which nurses' work patterns have changed over time. They provide a baseline to inform policy debate, and against which future interventions designed to change patterns of work might be measured.

## Competing interests

The authors declare that they have no competing interests.

## Authors' contributions

JW and CD conceptualised the study and secured funding. NC contributed to study design, method development and data collection. JW and LL designed the analysis strategy and LL applied and extended this. JW drafted the manuscript and all authors actively participated in critical review of the manuscript. All authors read and approved the final manuscript.

## Pre-publication history

The pre-publication history for this paper can be accessed here:

http://www.biomedcentral.com/1472-6963/11/319/prepub
